# Pathways in the Pathophysiology of Coronavirus 19 Lung Disease Accessible to Prevention and Treatment

**DOI:** 10.3389/fphys.2020.00872

**Published:** 2020-08-14

**Authors:** Michael Eisenhut, Jae Il Shin

**Affiliations:** ^1^Children’s & Adolescent Services, Luton & Dunstable University Hospital NHS Foundation Trust, Luton, United Kingdom; ^2^Department of Pediatrics, Yonsei University College of Medicine, Seoul, South Korea

**Keywords:** tumor necrosis factor, conductance regulator chloride channel, epithelial sodium channel, Na(+)/K(+)/2Cl(−) cotransporter type 1 cystic fibrosis transmembrane, furosemide, acute respiratory distress syndrome, acute lung injury, pulmonary edema

## Abstract

**Background:** In COVID 19 related lung disease, which is a leading cause of death from this disease, cytokines like tumor necrosis factor-alpha (TNF alpha) may be pivotal in the pathogenesis. TNF alpha reduces fluid absorption due to impairment of sodium and chloride transport required for building an osmotic gradient across epithelial cells, which in the airways maintains airway surface liquid helping to keep airways open and enabling bacterial clearance and aids water absorption from the alveolar spaces. TNF alpha can, through Rho-kinase, disintegrate the endothelial and epithelial cytoskeleton, and thus break up intercellular tight junctional proteins, breaching the intercellular barrier, which prevents flooding of the interstitial and alveolar spaces with fluid.

**Hypotheses:** (1) Preservation and restoration of airway and alveolar epithelial sodium and chloride transport and the cytoskeleton dependent integrity of the cell barriers within the lung can prevent and treat COVID 19 lung disease. (2) TNF alpha is the key mediator of pulmonary edema in COVID 19 lung disease.

**Confirmation of hypothesis and implications:** The role of a reduction in the function of epithelial sodium and chloride transport could with regards to chloride transport be tested by analysis of chloride levels in exhaled breath condensate and levels correlated with TNF alpha concentrations. Reduced levels would indicate a reduction of the function of the cystic fibrosis transmembrane conductance regulator (CFTR) chloride channel and a correlation with TNF alpha levels indicative of its involvement. Anti-TNF alpha treatment with antibodies is already available and needs to be tested in randomized controlled trials of COVID 19 lung disease. TNF alpha levels could also be reduced by statins, aspirin, and curcumin. Chloride transport could be facilitated by CFTR activators, including curcumin and phosphodiesterase-5 inhibitors. Sodium and chloride transport could be further regulated to prevent accumulation of alveolar fluid by use of Na(+)/K(+)/2Cl(−) cotransporter type 1 inhibitors, which have been associated with improved outcome in adults ventilated for acute respiratory distress syndrome (ARDS) in randomized controlled trials. Primary prevention of coronavirus infection and TNF alpha release in response to it could be improved by induction of antimicrobial peptides LL-37 and human beta defensin-2 and reduction of TNF alpha production by vitamin D prophylaxis for the population as a whole.

## Introduction

### Lessons From the Radiological Features of COVID 19 Lung Disease

A chest computed tomographic (CT) study in Wuhan, China of 131 COVID 19 patients showed that changes found were mainly bilateral peripheral ground glass opacities (GGO) combined with or without co-existing consolidations (total of 62% of cases; Li et al., 2020).

Another study of 73 cases demonstrated in the majority (*n* = 43) single or multiple GGOs in the periphery of the lungs. In the 21 patients with more severe clinical disease, extensive GGO and pulmonary consolidations were found in 16/21 and 5/21 cases, respectively. The authors commented that the changes were similar to those found in influenza H1N1 virus pneumonia (Liu et al., 2020).

A pictorial review of chest CT manifestations of COVID 19 lung disease summarized that the most common features is GGO, which in COVID 19 patients are commonly in a peripheral lung and subpleural distribution in up to 98% of patients. This is followed by consolidations, which are increasingly common with further progression of the disease (Ye et al., 2020).

### Lessons From Autopsy Results

The first detailed autopsy result of a 50-year-old man in Beijing with COVID 19 associated acute respiratory distress syndrome (ARDS), demonstrated at day 14 of illness, unequal appearances of both lungs with “bilateral diffuse alveolar damage” with “cellular fibromyxoid exudates” and features of pulmonary edema with formation of “hyaline membranes” (Xu et al., 2020).

In a study of 10 fatal cases, which was done in Sao Paulo, Brazil using ultrasound-based minimally invasive autopsies histological samples from lungs revealed “diffuse alveolar damage with intense epithelial viral cytopathic effects involving alveolar and small airway epithelium and little lymphocytic infiltration.” “A variable number of small fibrinous thrombi in small pulmonary arterioles in areas of both damaged and more preserved lung parenchyma” was noted in eight cases (Dolhnikoff et al., 2020). A subsequent autopsy study of seven patients revealed disseminated pulmonary vascular thrombosis more widespread than in influenza (Ackermann et al., 2020).

### Immunopathological Features of Severe COVID 19 Lung Disease

Twenty one patients with COVID-19 were analyzed with regard to features of their immunological response retrospectively. Compared with moderate cases, severe cases had significantly elevated concentrations of TNF alpha, interleukin (IL)-2R, IL-6, and IL-10 (Chen et al., 2020b).

The action of TNF alpha can explain the multi-organ failure found in severe COVID 19 disease. It is a vasoconstriction causing cytokine which can cause ischemia in all organ systems (Vila and Salaices, 2005), including heart, liver, and kidneys as seen in fatal cases (Chen et al., 2020a), thus responsible for widespread ischemic organ damage in multiple organs. Radiological and autopsy findings are consistent with an inflammatory process causing pulmonary interstitial and alveolar fluid accumulation.

### The Hypotheses

- Preservation and restoration of airway and alveolar epithelial sodium and chloride transport and the cytoskeleton dependent integrity of the cell barriers within the lung can prevent and treat COVID 19 lung disease.
- TNF alpha is the key mediator of pulmonary edema in COVID 19 lung disease.

## Explanation of the Hypotheses

### The Role of Epithelial and Endothelial Sodium and Chloride Transport in Pulmonary Airway Liquid Film Depth and Alveolar Fluid Clearance

The depth of the airway liquid film is dependent on uptake of sodium through epithelial sodium channels (ENaC) and secretion of chloride through the cystic fibrosis transmembrane conductance regulator (CFTR) chloride channel in airway epithelial cells. In genetic mutations, inactivating ENaC (systemic pseudohypoaldosteronism) or CFTR (cystic fibrosis), there are tenacious dehydrated airway secretions causing lung disease and this is the clinical picture found in COVID 19 patients regarding their airway secretions. ENaC and CFTR dysfunction in alveolar epithelial cells on the other hand have been linked to pulmonary edema due to reduced alveolar epithelial sodium and chloride absorption, required to establish the osmotic gradient for lung water absorption from the alveolar space through aquaporin channels and para-cellular pathways. CFTR is hereby a key regulator of alveolar liquid absorption. CFTR promoted liquid clearance *in vitro* and alveolar liquid clearance *ex vivo* (Matthay et al., 2005; Mutlu et al., 2005; Fang et al., 2006; Li et al., 2012). Inflammation involving the cytokine TNF alpha can cause ENaC and CFTR and epi‐ and endothelial barrier dysfunction and is a dominant feature of COVID-19 induced ARDS. TNF alpha production has been found to be increased by action of the spike protein in the related SARS coronavirus 1 through shedding of the angiotensin converting enzyme-2 (ACE-2) ectodomain, a process which mediated subsequently by the cytoplasmic tail of ACE-2 activates the TNF alpha converting enzyme leading to increased TNF production (Haga et al., 2008).

### The Link Between TNF alpha and Excessive Lung Water

#### TNF alpha and the Cytoskeleton

The lung capillary endothelial and alveolar epithelial barrier function depends on the integrity of the cytoskeleton, components of which are actin-based microfilaments, microtubules, and intermediate filaments. TNF alpha induces endothelial actin microfilament disruption and intercellular gap formation that determine transcellular permeability. TNF alpha has been demonstrated to cause microtubule destabilization in human pulmonary artery endothelial cells (EC). TNF alpha hereby induced disassembly of the peripheral microtubule network. This is associated with TNF alpha–induced increases in permeability of EC layers (Petrache et al., 2003).

TNF alpha induced microtubule re-arrangement together with an activation of EC contraction and permeability increase *via* G-protein coupled receptor mediated p38–mitogen-activated protein kinase signaling and Rho-kinase activated phosphorylation of MLC phosphatase (MYPT1) leading to MYPT1 inactivation. This inhibition of phosphatase resulted in accumulation of diphospho-myosin light chains, which lead to acto-myosin polymerization, resulting in actomyosin contraction through stress fiber formation. This induced endothelial and epithelial cell contraction and therefore intercellular gap formation leading to permeability increase (Birukova et al., 2004; Kása et al., 2015).

#### TNF alpha and Pulmonary Ion Transport

We previously described *in vivo* the association of altered epithelial chloride transport in patients with reversible pulmonary edema associated with severe meningococcal septicemia, which like COVID 19 lung disease is characterized by a “cytokine storm” (Eisenhut et al., 2006), which is also characterized by high levels of the pro-inflammatory cytokine TNF alpha (Van Deuren et al., 1995).TNF alpha can inactivate ion transport draining alveolar and interstitial fluid as explained previously (Eisenhut and Wallace, 2011; Peteranderl et al., 2017). Pointers toward the underlying mechanisms are that incubation of alveolar epithelial cells with TNF alpha (24 h) seemed to reduce epithelial sodium channel (ENaC) mRNA expression (Dagenais et al., 2004; Yamagata et al., 2009). Epithelial sodium channels allow for build-up of the osmotic gradient of sodium absorbing alveolar liquid. TNF alpha reduced the expression of alpha, beta, and gamma-subunit mRNA of ENaC. TNF alpha treated cells displayed a reduction in alpha ENaC mRNA stability. *In vivo* studies in patients with meningococcal septicemia induced pulmonary edema however did not show a reduced sodium transport but features of an inactivation of the CFTR chloride channel, which is another essential prerequisite for removal of alveolar liquid by osmosis. This was in keeping with the previous *in vitro* data on the action of TNF alpha on CFTR chloride channels (Nakamura et al., 1992)

In the colonic epithelium-derived tumor cell line (HT-29), it was established that TNF alpha reduced the stability of CFTR mRNA transcripts by a 35% shortening of its half-life. Future research needs to establish whether as demonstrated in influenza A virus induced pulmonary edema it is, in addition to a dysfunction of the epithelial sodium channel possibly not a reduction but an activation of the CFTR (Wolk et al., 2008; Peteranderl et al., 2017), which is key in the pathogenesis e.g., *via* chloride secretion into the airway drawing water by osmosis with it.

## Evidence to Support the Hypothesis

TNF is central in the pathogenesis of inflammation and triggers the release of a multitude of inflammatory mediators including IL-1, IL-6, IL-8, and granulocyte/macrophage colony stimulating factor (GMCSF; Fiers, 1991; Szatmary, 1999), which have been found to be elevated in COVID-19 lung disease. IL-1 itself, which has the potential to derange epi‐ and endothelial barrier integrity and alveolar ion transport similar to TNF (Eisenhut and Wallace, 2011), has not been found to be significantly higher in patients with more severe COVID 19 lung disease (Qin et al., 2020). IL-6, IL-8, and GMCSF have not previously been directly linked to the pathogenesis of deranged ion transport in ARDS but there is limited evidence of the effects of IL-6 in reduction of endothelial barrier integrity *in vitro* and IL-8 in involvement of smoke inhalation induced lung injury in the rabbit model (Laffon et al., 1999; Birukova et al., 2016).

Early COVID 19 airway disease is characterized by dry, sticky airway secretions leading to collapse of lung segments (atelectasis) and a dry cough. This previously prompted research groups in China to set up a trial of acetylcysteine inhalation for mucolytic effect *via* the tracheal tube in intubated patients (now abandoned; Lythgoe and Middleton, 2020). This phenomenon is consistent with a reduction of CFTR mediated airway liquid film in CFTR dysfunction. Clinical, chest CT and pathological findings in COVID 19 lung disease are consistent with a combination of reduced airway liquid film causing tenacious airway secretions, atelectasis and consolidation, and pulmonary edema from reduced alveolar fluid clearance. Alveolar fluid clearance is impaired in inflammation related pulmonary edema and ARDS and fluid clearance is significantly lower in patients who die of ARDS (Ware and Matthay, 2001).

### Risk Factors for COVID-19 Lung Disease

Advanced age and obesity have both been identified as risk factors for severe COVID-19 lung disease (Garg et al., 2020; Toussie et al., 2020). Both have been associated with an increase in TNF alpha production (Huang et al., 2005; Kern et al., 2018)

### TNF alpha and Coagulopathy

Injection of TNF into healthy volunteers induces a pro-coagulant state (Thijs et al., 1993). The underlying mechanisms involve down-regulation of thrombomodulin at an endothelial level, reduction of tissue plasminogen and an increase in procoagulant factors like platelet activating factor and tissue plasminogen activator inhibitor (Aderka, 1991). This may explain the pulmonary thrombosis discovered in COVID 19 lung disease as outlined above.

### Direct Coronaviral Effects on ENaC and CFTR Function

#### Epithelial Sodium Channel

Previous *in vitro* studies of expression of the related SARS-coronavirus 1 proteins in *Xenopus* oocytes and human airway epithelial cells showed that co-expression of either SARS coronavirus 1 S or E protein alongside ENaC subunits significantly decreased amiloride-sensitive Na^+^ currents and γ-ENaC protein levels. S and E proteins acted through a reduction of ENaC exocytosis. Inhibition of PKCα/β1 and PKCζ restored the downregulation of ENaC activity by SARS. These results supported the hypothesis that pulmonary edema associated with SARS coronavirus 1 infection could be related to activation of protein kinase C (PKC) by SARS proteins which then decrease ENaC presence and activity on pulmonary epithelial cells (Ji et al., 2009).

In addition, it is likely that double-strand RNA functioning as replicative intermediates during coronavirus infection (Hagemeijer et al., 2012) are – like postulated in influenza and respiratory syncytial virus (RSV) lung disease – involved in ENaC dysfunction. It has been demonstrated in *in vitro* investigations for influenza virus and RSV that they can inhibit amiloride-sensitive sodium transport in cells of the respiratory tract. This effect was found to occur through nucleotide/P2Y purinergic receptors demonstrated by use of the synthetic double-stranded RNA analog poly-inosinic-cytidylic acid to be probably mediated by double-strand RNA replication intermediates, which are TLR-3 ligands (Aeffner et al., 2011).

#### Cystic Fibrosis Transmembrane Conductance Regulator

A direct interaction of coronaviruses with CFTR has not been documented *in vitro* or *in vivo* but postulated from sequence data on several coronaviruses which revealed through alignments the main 3CL proteinase cleavage sites in polyproteins and this included some in the CFTR gene. This was obtained through use of a neural network able to recognize the cleavage sites in genomes (sensitivity 87.0%, specificity 99.0%; Kiemer et al., 2004).

## Evidence Against the Hypothesis Regarding TNF Alpha as the Key Mediator Causing Pulmonary Edema in COVID 19 Lung Disease

Animal studies using the rat model of *Pseudomonas aeruginosa* lung disease demonstrated that TNF alpha has the potential to increase alveolar fluid clearance (Rezaiguia et al., 1997), an effect which has been hypothesized to be linked to the lectin-like domain of TNF, which was synthesized as TIP peptide (Yang et al., 2010). The effect of this domain in the rat model was found to involve activation of the epithelial sodium channel *in vitro*: TIP peptide was shown to do this by binding to the carboxy-terminal domain of the α-subunit of the channel facilitating opening of the channel (Czikora et al., 2014). This led to clinical trials in an attempt to improve alveolar fluid clearance using this synthetic TIP peptide (Aigner et al., 2017; Krenn et al., 2017). Experiments in the rat model *in situ* and *ex vivo* demonstrated that the effect of this lectin-like domain as a part of the complete TNF molecule is only active if the binding of TNF to its receptor TNFR1 is inhibited. The TNFR1 mediated reduction in alveolar fluid clearance, thus appears to outweigh the effects of the lectin-like domain (Braun et al., 2005).

## Testing of the Hypothesis

The role of a reduction in function of epithelial sodium and chloride transport could be tested for chloride transport by analysis of chloride levels in exhaled breath condensate (EBC; Zacharasiewicz et al., 2004) and levels correlated with TNF alpha concentrations.

To accomplish this task, fluid in the filter reservoir of the outgoing tubing of ventilators can be analyzed for sodium and chloride levels compared to controls without pulmonary edema and corrected for dilution/evaporation by calculating the ratio to a reference molecule like urea: if the chloride levels are increased, a predominant hyperactivation of CFTR is likely involved and if levels are found to be reduced, a reduction of CFTR function is likely. The same applies to conclusions for sodium levels on ENaC function. The possible involvement of TNF alpha can equally be analyzed by measurement of TNF alpha in EBC *via* an ELISA and a negative correlation of chloride or sodium levels with TNF alpha levels would confirm an involvement in CFTR or ENaC dysfunction.

## Implications of a Confirmation of the Hypothesis

### Potential Avenues for Treatment and Prevention

#### Anti-TNF alpha Treatments

The coronavirus mouse model demonstrated that the TNF alpha pathway is a key in the pathogenesis of systemic murine coronavirus disease: infection with SARS-CoV 1 of double-null TNF alpharsf1a/1b−/− mice showed that the mouse strain used had a reduced weight loss, indicating that TNF alpha may promote pathogenesis in SARS-CoV 1 disease mediated by TNF alpha receptors (McDermott et al., 2016). Administration of anti-TNF alpha antibodies reduced weight loss and increased survival in a SARS-CoV 1 pneumonia mouse model (Channappanavar et al., 2016). We support the appeal by others for anti-tumor necrosis factor therapies for COVID-19 (Feldmann et al., 2020).

Implementation of anti-TNF alpha therapies in patients with ARDS may be too late as the cell barriers and ion transport systems may already be irreparably disrupted. Prevention of TNF alpha production in patients at risk may prevent hospital admission and deterioration of infected patients at risk of ARDS from this infection.

Two systematic reviews of randomized placebo controlled trials revealed anti-TNF antibody therapies as used to treat auto-immune diseases have been associated with a significantly increased risk of infections ranging from 20 (any infection) to 250% (tuberculosis; Minozzi et al., 2016). In another systematic review, the number needed to harm from serious infections (infection that requires antimicrobial therapy and/or hospitalization) was 59 (95% confidence interval (CI), 39–125) and a pooled odds ratio (OR) for malignancy was 3.3 [95% CI, 1.2–9.1] with a number needed to generate one additional malignancy 154 (95% CI, 91–500) within a treatment period of 6–12 months (Bongartz et al., 2006). This highlights the need to develop strategies that preferentially blunt the deleterious but not the positive actions of the cytokine against infections and malignancies.

In the following, we are exploring preventative options, which could reduce TNF alpha production without the adverse effects on infection rates and malignancy observed with anti-TNF antibody therapies.

#### Steroids

Steroids suppress cytokine production, including TNF alpha but have significant adverse effects which may outweigh any benefit; there are no published prospective double blind randomized controlled trials (RCT) on steroid in COVID 19 lung disease but a meta-analysis of 15 mainly retrospective cohort and studies using historical controls showed that in 5,270 patients analyzed corticosteroid treatment was associated with higher mortality (RR = 2.11; 95% CI = 1.13–3.94) possibly related to a higher rate of bacterial infection and hypokalemia (Yang et al., 2020). This lack of an effect is likely due to use after the effects of TNF alpha on tissues is already established in patients in the intensive care unit. However, this meta-analysis should be interpreted with caution because a high mortality rate shown in corticosteroid-treated group in SARS and Middle East respiratory syndrome (MERS) could be due to more systemic compromise evident from baseline characteristics (e.g., older age, higher rate of comorbid conditions, and severe patients) in most individual studies.

#### Statins

Animal model data showed that reduction of inflammation as mediated by NF-kappaB in SARS coronavirus-infected mice increased survival (DeDiego et al., 2014) and atorvastatin attenuated TLR4-mediated NF-kappaB activation (Chansrichavala et al., 2009). To asses this potential, which would apply to COVID 19 lung disease, it is important to look at evidence from treatment of conditions leading to activation of NF-kappaB like bacterial infections and then ALI/ARDS by statins. In a double blind randomized controlled trial, the administration of simvastatin in bacterial infections was investigated. Enrolled were a total of 83 patients and 42 patients received simvastatin and 41 received placebos. In the simvastatin group, TNF alpha and IL-6 levels were reduced (Novack et al., 2009). In animal studies (mice), simvastatin attenuated vascular leak and inflammation in inflammatory lung injury in mice (Jacobson et al., 2005). If administered in already present acute lung injury (ALI) statin administration in humans did not alter the outcome with regard to organ failures (Kor et al., 2009) in one study. A subsequent systematic review and meta-analysis of both randomized clinical trials and cohort studies, which included a total of 12 studies (eight cohort studies and four randomized controlled trials with a total of 9,309 patients), which did not analyze the effects of statin use commenced only during ALI/ARDS management separately, found that the sepsis-related organ failure assessment (SOFA) was significantly lower in patients receiving statins and the number of ventilator-free days was increased among statin users (Feng, 2018). A recent re-analysis of one randomised controlled trial (RCT) using statins as an intervention to treat ALI/ARD, which was included in this meta-analysis, focused on a subgroup of patients with higher inflammatory markers. This included higher values of sTNRr-1 and IL-6 levels. The hyper-inflammatory subphenotype had fewer ventilator-free days. As opposed to the result of the original trial report, which did not identify a difference in 28-day survival between simvastatin and placebo, this analysis comparing subjects in a hypo-inflammatory and hyper-inflammatory subphenotype found that within the hyper-inflammatory subphenotype simvastatin treated patients had a significantly reduced 28‐ and 90-day mortality (*p* = 0.008). The mortality was 32% in the hyper-inflammatory subphenotype treated with simvastatin in comparison to 45% in the placebo group with this subphenotype (Calfee et al., 2018).

Analysis of a prospective cohort study in 575 critically ill patients those on statin therapy prior to hospitalization had a reduced probability of developing ALI/ARDS (OR, 0.60; 95% CI, 0.36–0.99; O’Neal et al., 2011).

In a 3-year prospective analysis of data 11,490 patients with atherosclerotic diseases, a comparison of two groups‐ those receiving statins in the final month of follow-up and those who did not receive statins revealed the following: compared to the control group, the risk of infection-related mortality was significantly lower in the statin group [0.9% vs. 4.1%, relative risk of 0.22 (95% CI, 0.17–0.28)]. This result remained highly significant in a survival analysis, which took potential confounders into account and used a propensity score for receiving statins (hazard ratio, 0.37; 95% CI, 0.27–0.52; Almog et al., 2007).

#### Aspirin

Aspirin reduces inflammatory mediator production, including TNF alpha and IL-6 (Loesche et al., 2012). Erlich et al. (2011) investigated the association of prehospital aspirin therapy and ALI/ARDS. Included were 161 patients with at least one major risk factor for ALI/ARDS. Therapy with aspirin on admission was associated with a significantly lower rate of ALI/ARDS when compared to patients without aspirin (17.7% vs. 28.0%; OR, 0.37; 95% CI, 0.16–0.84). The authors reported that the benefit of aspirin therapy remained significant after adjusting for smoking and pre-hospital statin use but not for coronary heart disease, which was more common in the group using aspirin (Erlich et al., 2011). In another observational study on the association of pre-hospital aspirin therapy and ALI/ARDS (Kor et al., 2011) on 3,855 patients out of which 25% were receiving aspirin at the time of hospitalization patients with aspirin were more severely affected as evident from higher APACHEII scores [12 (8–16) vs. 9 (5–14)]. There was a lower incidence of ALI/ARDS in patients on aspirin (OR, 0.65; 95% CI, 0.46–0.90). In another such study, pre-hospital use of both statins and aspirin was associated with the lowest rates of ALI/ARDS and mortality (O’Neal et al., 2011). Future research in COVID-19 lung disease needs to establish whether a reduction in pulmonary vascular thrombosis is involved in this aspirin effect.

#### Curcumin

Curcumin reduces TNF alpha production through epigenetic modulation of TNF alpha producing mononuclear blood cells (Sahebkar et al., 2016; Hassan et al., 2019). In a meta-analysis including eight RCTs, the results demonstrated a significant reduction of circulating TNF alpha levels following curcumin administration (WMD, −4.69 pg/ml; 95% CI, −7.10, −2.28; *p* < 0.001). This effect size was robust in sensitivity analysis.

#### Curcumin and CFTR

CFTR function can be enhanced by curcumin (Becq et al., 2011). Curcumin hereby facilitates the release of F508-CFTR from the endoplasmic reticulum, thus aiding integration in the plasma membrane. After integration, it enhances channel activity. This appears to be related to the disintegration of the calnexin-F508-CFTR complex and a stabilizing effect on the tertiary structure of F508-CFTR.

The fact that unencapsulated curcumin is hardly absorbed makes it necessary to use nanoparticle preparations like those encapsulated in poly lactic-co-glycolic acid nanoparticles.

#### Phosphodiesterase 5 Inhibitors

Trials in human CF nasal epithelial and baby hamster kidney (BHK) cells and mouse experiments showed that the phosphodiesterase 5 (PDE5) inhibitor sildenafil restored F508del-CFTR activity and activated chloride transport on nasal potential difference measurement in mice *in vivo*. In another mouse strain, it was associated with an improved CFTR-mediated current attributable to a reduction of an excessive proinflammatory response (Becq et al., 2011).

#### Ivacaftor

Ivacaftor can hold the channel structure of CFTR open, thus facilitating chloride flow. This then normalizes the amount of fluid at the surface of the cell. Ivacaftor significantly enhanced pulmonary fluid clearance in isolated pig lung lobes. In a model using model elevated hydrostatic pressure induced pulmonary edema, which resulted in decreased CFTR activity and liquid absorption, ivacaftor partially reversed this pathology (Li et al., 2017).

#### Vitamin D

As reviewed recently (Greiller and Martineau, 2015), vitamin D has through the induction of an increased production of anti-microbial peptides LL-37 and beta-defensin-2 in monocytes broad spectrum anti-viral effects.

With regard to coronaviruses, vitamin D induced beta defensin 2 promoted antiviral immunity *in vitro* and *in vivo*. This was demonstrated by means of a receptor-binding domain (RBD) of Middle East respiratory syndrome-coronavirus (MERS-CoV) spike protein (S RBD). When HBD 2-conjugated S RBD was incubated with THP-1 human monocytic cells, the expression of IFN-β, IFN-γ, MxA, PKR, and RNaseL molecules was increased compared to controls (Kim et al., 2018).

In addition, it has immunomodulatory effects reducing TNF alpha production. In alveolar A549 cells, influenza infection increased the production of pro-inflammatory cytokines and chemokines and the addition of vitamin D either before or after influenza infection reduced gene expression of among other cytokines TNF alpha and IL-6.

Most of the numerous observational studies on the association between vitamin D status and acute respiratory tract infections (RTI) showed independent associations between low vitamin D status and increased risk of acute viral respiratory tract infections. (Greiller and Martineau, 2015). A meta-analysis of 11-placebo controlled studies using vitamin D for the prevention of RTI (*n* = 5,660) demonstrated a protective effect against RTI (OR, 0.64; 95% CI, 0.49–0.84; Bergman et al., 2013).

With regard to effects of vitamin D on the severity of ARDS, there are two low quality retrospective cohort studies and both show an association of low vitamin D levels with more severe ARDS of a variety of causes: in 1985, critically ill adults patients with 25(OH)D levels lower than 30 mg/ml had a significantly higher risk of acute respiratory failure (Thickett et al., 2015). In another study, which included 52 patients with ARDS, 57 patients who had esophagectomy (at risk of ARDS) and 8 patients who had esophagectomy with high-dose vitamin D supplementation prior to surgery, the odds of ARDS in patients with 25(OH)D_3_ < 20 nmol/L was 3.5-fold higher than that of patients with 25(OH)D_3_ ≥ 20 nmol/L [OR = 3.5 (95% CI, 1.06–11.6; *p* = 0.040)]. After adjustment for age, gender, diagnostic category, staging, and degree of cigarette consumption, patients with 25(OH)D_3_ < 20 nmol/L show a significantly higher odds of ARDS compared to patients with 25(OH)D > 20 nmol/L [OR = 4.2 (95% CI, 1.13–15.9; *p* = 0.032)]. On logistic regression analysis, 1 nmol/L increments of 25(OH)D were associated with reduction in odds of ARDS by 17% for every 1 nmol/L. On admission to ITU lower plasma 1, 25(OH)_2_D levels were found in patients who died compared to survivors (Dancer et al., 2015).

#### Treatment of Established ARDS With NKCC1 Inhibitors

In pulmonary edema caused by inflammation, ENaC inhibition generates a gradient for Na+ influx due to lowered intracellular sodium concentrations. This influx is regulated by the basolateral Na(+)/K(+)/2Cl(−) cotransporter type 1(NKCC1) in alveolar type 1 epithelial cells. Cl− enters in cotransport with Na+, and can leave the cell along an electrochemical gradient on the apical side through CFTR, resulting in Cl−-driven fluid secretion and subsequently alveolar liquid accumulation as seen in pulmonary edema and ARDS (Weidenfeld and Kuebler, 2017), and TNF alpha upregulates NKCC1 mRNA and protein levels. In the largest (*n* = 1,000) and highest quality randomized controlled trial comparing conservative with liberal fluid management in ARDS, on which current fluid management guidelines globally are based, patients in the conservative arm of the study received significantly more often (133/497 vs. 312/503) and higher (74 vs. 148 mg/day) daily doses of furosemide which is an inhibitor of the NKCC1 (National Heart, Lung, and Blood Institute Acute Respiratory Distress Syndrome (ARDS) Clinical Trials Network et al., 2006). NKCC1 inhibition may have made the crucial difference in outcome including that it was associated with more ventilator-free days (14.6 ± 0.5 vs. 12.1 ± 0.5, *p* < 0.001) and days outside the intensive care unit (13.4 ± 0.4 vs. 11.2 ± 0.4, *p* < 0.001) in this “conservative” treatment group. This is supported by recent experimental data (Shen et al., 2018). Despite an approval of the conservative fluid management strategy, recent guidelines on COVID 19 induced ARDS management (Alhazzani et al., 2020; Matthay et al., 2020) do not mention furosemide or other NKCC1 inhibitors like bumetanide. It appears to be regarded as sufficient to just drain a certain amount of fluid from the patient by measures including hemodialysis. This ignores the specific potentially beneficial effect of alveolar epithelial NKCC1 inhibition by furosemide and other loop diuretics on lung fluid clearance and needs to be immediately addressed by randomized controlled trials.

## Conclusion

Strategies for prevention of COVID 19 lung disease in vulnerable groups need to consider vitamin D, aspirin, statins, and curcumin as primary prevention of the potentially fatal hyperinflammatory state leading to acute lung injury, ARDS, and multi-organ failure for all members of those groups and vitamin D for the population as a whole.

For treatment of established acute lung injury or ARDS due to COVID 19, NKCC1 inhibitors, anti-TNF alpha treatment, aspirin, and statins need to be included in prospective randomized placebo controlled trials.

## Data Availability Statement

The original contributions presented in the study are included in the article/supplementary material and further inquiries can be directed to the corresponding author.

## Author Contributions

Both authors contributed to the article and approved the submitted version.

### Conflict of Interest

The authors declare that the research was conducted in the absence of any commercial or financial relationships that could be construed as a potential conflict of interest.
